# Removal of a Double-Pointed Needle from the Submaxillary Connective Tissue, by the Aid of Manipulation

**Published:** 1881-09

**Authors:** Samuel Kohn

**Affiliations:** New York


### REMOVAL OF A DOUBLE-POINTED NEEDLE FROM THE SUBMAXILLARY CONNECTIVE TISSUE, BY THE AID OF MANIPULATION.

BY SAMUEL KOHN, M.D.,
NEW YORK.

Miss M. L——, aged seventeen, presented herself in the Throat Class of the New York Dispensary, June 1st, with the following history: She is employed in running a patent embroidering machine; while changing the needles on the morning of the above date, she placed one in her mouth until such time as its use might be required. The needles, one of which she brought to me as a sample, are short, being only about three-quarters of an inch in length, sharp at both ends, with the eye placed midway between the two points. She states that, soon after taking the needle into her mouth she felt a sharp pricking sensation near the root of the tongue, and that the needle disappeared, working its way into the sublingual connective tissue. Two physicians who were successively consulted, examined the mouth, and not finding any evidence of the truthfulness of the girl's statement, declared that she either swallowed the needle or that she was mistaken. On examining the floor of the mouth carefully, I found, opposite the buccal side of the second molar tooth, a minute perforation of the mucous membrane, surrounded by a narrow white rim of superficial ulceration; this the girl declared to be the point at which the needle entered. With two fingers in the floor of the mouth, and external counter-pressure, the position of the foreign body could not be localized. Thereupon, the unsharped end of a lead pencil was pressed against various points of the right submaxillary region, and the patient told to say when the point of the needle pricked the tissue; she finally felt a piercing pain in the sub-parotid region. Bimanual manipulation—two fingers of the right hand in the floor of the mouth, and three fingers of the left, at this point externally continued for over half an hour, finally succeeded in bringing the point through the mucous membrane opposite the buccal side of the second bicuspid tooth; but not enough of the needle appearing to offer a hold, I was compelled to release the grasp I had of the tissues and obtain other finer instruments. On returning to the patient, it was found that owing to several deglutitiory movements, the needle had again disappeared. Bimanual manipulation for over half an hour again succeeded in bringing through the mucous membrane of the floor of the mouth the point of the needle in the same place as before. Dr. Valentine, the house physician, was now called to my assistance, and while I pressed with full force upward and inward against the right submaxillary tissue, Dr. Valentine, with a pair of polypus forceps, attempted to seize the point of the needle, and after several trials succeeded in extracting this troublesome foreign body. My apology for sending this case to the Record lies, 1st, in its uniqueness; 2d, in the evidence it affords of the value of a careful examination and of manipulation. Had a cutting operation for the recovery of this delicate foreign body been performed, the chances are, I venture to say, against the probability of its having been found, not to speak of the many unpleasantnesses attending such an operation.—*The Medical Record, N. Y.*

